# Highly efficient *in vitro* regeneration, establishment of callus and cell suspension cultures and RAPD analysis of regenerants of *Swertia lawii* Burkill

**DOI:** 10.1016/j.btre.2015.03.003

**Published:** 2015-03-11

**Authors:** Parthraj R. Kshirsagar, Jaykumar J. Chavan, Suraj D. Umdale, Mansingraj S. Nimbalkar, Ghansham B. Dixit, Nikhil B. Gaikwad

**Affiliations:** a Department of Botany, Shivaji University, Kolhapur 416 004, India; b Department of Botany, Yashavantrao Chavan Institute of Science, Satara 415 001, India; c Department of Biotechnology, Yashavantrao Chavan Institute of Science, Satara 415 001, India

**Keywords:** Callus, Cell suspension, Micropropagation, Medicinal herb, RAPD, *Swertia lawii*

## Abstract

Highly efficient *in vitro* regeneration system has been developed for *Swertia lawii* Burkill, an important herb used as substitute for *Swertia chirayita*. Shoot tips explants were cultured on MS medium with various phytohormones for multiple shoot production. The best shoot production frequency (100%) and maximum shoots (10.4 ± 0.8) were obtained on MS media containing TDZ (3.0 mg l^−1^) in combination with IBA (0.3 mg l^−1^). Maximum callus induction (95 ± 4.8%) and callus growth (1.7 ± 0.4 gm) was achieved on MS medium with 2, 4-D (3.0 mg l^−1^). Cell suspension cultures were established and studied for their growth kinetics. Shoots were rooted best (22.1 ± 2.5) in 1/2 MS medium with IAA (3.0 mg l^−1^). The genetic uniformity of the micropropagated clones was assessed using RAPD markers. Out of 405 bands, 400 (98.76%) were monomorphic and rest 5 (1.24%) were polymorphic. High multiplication frequency and low risk of genetic instability ensures the efficacy of this protocol.

## Graphical abstract

**Figure:**
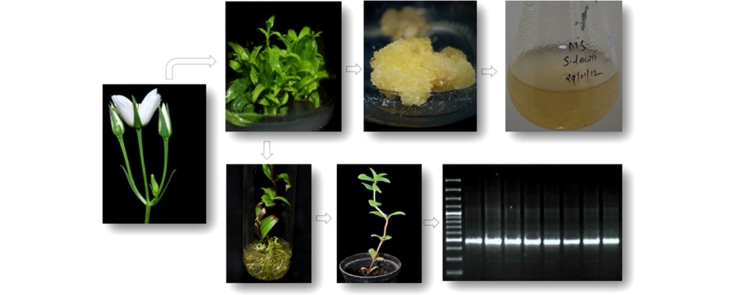

## 1 Introduction

**Fig. 1:**
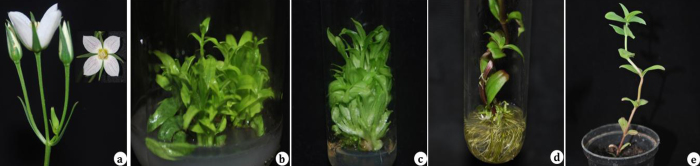
Micropropagation of *Swertia lawii*. a: Flowers, b and c: shoot multiplication (MS + 3.0 mg l^−1^ TDZ + 0.3 mg l^−1^ IBA), d: *in vitro* rooting (2.0 mg l^−1^ IBA), e: hardened plant.

*Swertia* L., belongs to the family Gentinaceae, is a group of multipurpose medicinal herbs used in the Indian and Chinese traditional system of medicine since prehistoric time. About 40 species are reported to be available in the eastern and western parts of Indian Himalayas at high altitudes, while Western Ghat harbors about more than eight *Swertia* species [1]. The genus is a rich source of xanthonoids, flavonoids, iridoids and terpenoids [1]. The extracts of a number of *Swertia* species have been used in folk medicine for the treatment of hepatitis, cholecystitis, pneumonia, dysentery, scabies, spasm, pain and neurasthenia [2]. The herbs are extensively used as bitter tonic and febrifuges in ayurvedic system of medicine. Isolated bioactive compounds and various extracts of *Swertia* species possess several pharmacological properties [2], [3], [4]. Due to the high medicinal implications of this genus, many species has an established domestic and international market which is increasing at a rate of 10% annually [1], [5]. Among all the species, *Swertia chirayita* was the most investigated species in terms of phytochemical analysis and pharmacognosy [4], [6]. Almost *Swertia* species (including *Swertia lawii*) are rich source of oleanolic acid and ursolic acid, two pharmacologically important compounds [7], [8], [9]. *S. lawii* is an important medicinal herb which is used as adulterant and also as substitute to *S. chirayita* [10], [11], [12]. Several xanthone compounds were isolated from various organs of *S. lawii* and it was found that it is rich source of erythrocentaurin, an important bioactive compound [10], [13]. Apart from its medical implications, the species has ornamental flowers (Fig. 1a).

Problem associated with *in vitro* plant regeneration is occurrence of somaclonal variations among the sub-clones of parental line, arising as a direct outcome of *in vitro* culture of plant cells, tissue and organs [14], [15]. These genetic defects in the regenerants limit the utility of plant tissue culture techniques for large-scale multiplication. However, in recent year’s molecular marker techniques such as RAPD and ISSR plays a significant role for detecting the genetic variation in the regenerants.

The *in vitro* propagation studies in *Swertia* species are limited with *S. chirayita* and few other species [16], [17], [18], [19]. The establishment of tissue culture protocol will be an important action for multiplication, germplasm conservation and secondary metabolite production in *S. lawii*. Thus, the objective of this work was to systematically study new strategies for *in vitro* culture of *S. lawii* aiming at developing efficient plant propagation protocol as well as establishing callus and cell suspension cultures. Moreover, the genetic stability among *in vitro* raised clones was assessed by RAPD markers.

## 2 Experimental

### 2.1 Plant material and culture conditions

Mature fruits of *S. lawii* were collected from Panhala locality of Western Ghats. Seeds were separated and washed with sterile distilled water in vials for 2–3 times. Then the seeds were surface disinfected with aqueous solution of 0.1% HgCl_2_ for 2 min and finally washed with sterile distilled water for 2–3 times. For *in vitro* germination, seeds were inoculated on the Murashige and Skoog (MS) medium with vitamins, sucrose (3%, w/v) and solidified with 0.2% clarigel (Himedia, India). Before autoclaving at 121 °C for 15 min, the pH of the medium was adjusted to 5.8. All the cultures were maintained at 25 ± 1 °C with photoperiod of 16-h using a photosynthetic photon flux density (PPFD) of 40 μmol m^−2^ s^−1^ provided by cool white fluorescent lamps (Philips, India) for 30 days.

### 2.2 Seed germination, primary cultures and shoot multiplication

Surface sterilized seeds were cultured on MS basal medium for *in vitro* germination. In order to obtain cultures, shoot apices were excised from 30 day old seedlings were inoculated on MS medium supplemented with 0.5 mg l^−1^ BAP and incubated under a 16-h photoperiod. In order to optimize shoot multiplication, shoot tips were excised from primary cultures were cultured on MS medium supplemented with different concentrations and combinations of plant growth regulators (BAP, KN, TDZ and IBA). Sub culturing was done once at 4 weeks of interval.

### 2.3 Optimization of callus and cell suspension cultures

To induce callus, leaf explants were cultured on MS medium supplemented with different concentrations of 2, 4-D (1.0–5.0 mg l^−1^). Cultures were incubated at 25 ± 2 °C and 16 h photoperiod under 40 μmol m^−2^ s^−1^ photosynthetic photon flux density. To proliferate, callus was transferred to same media composition in which callus was induced. Callus induction response (%) and fresh callus weight (gm) were recorded for each concentration of 2, 4-D. Cell suspension cultures were established from friable calluses obtained from optimal 2, 4-D concentration (2.0 mg l^−1^). Two milliliter of packed callus cells was transferred to 150 ml conical flask containing 20 ml of liquid MS medium with different concentrations of 2, 4-D, BAP and glutamine (200 mg l^−1^) per flask. Flasks were closed with two layers of aluminum foil and incubated on orbital shaker (100 rpm) at 25 ± 1 °C in light (16 h photoperiod) for 30 days. The flasks were supplied with fresh medium after every 4 weeks. The cells were separated by centrifugation from full grown cultures, liquid medium was removed and fresh weight of cells was recorded. The callus mass or pellets were dried at 60 °C for 48 h in oven and weighed for their dry weight.

### 2.4 Rooting and acclimatization of regenerants

Elongated shoots with 2–3 pairs of healthy leaves were excised and transferred to rooting medium. The shoots were cultured on MS medium supplemented with various auxins *viz*. IBA (1.0–5.0 mg l^−1^), IAA (1.0–5.0 mg l^−1^) and NAA (1.0–5.0 mg l^−1^). Plantlets with well-developed shoots and roots were removed from the culture vessels and gently washed under running tap water. Roots were then treated with Bavistin (0.7%) before placing into plastic pots filled with sterile soil, sand and coco peat (1:2:1). The plantlets covered in transparent polyethylene bags were irrigated with sucrose free MS liquid salts for 8–10 days and were kept in culture room at 25 ± 1 °C with 16 h photoperiod and 40 μmol m^−2^ s^−1^ of irradiation. The bags were gradually removed and plantlets were transferred to large earthen pots containing garden soil. Survival rate in acclimatization was monitored after 2 months transfer to natural conditions.

### 2.5 RAPD analysis

DNA was isolated from fresh young leaves of donor plant as well as seven randomly selected *in vitro* raised plants. The genomic DNA was isolated following the protocol of Doyle and Doyle [21] with a little modification. Quality and quantity of genomic DNA was assessed by 0.9% agarose gel electrophoresis with λ uncut DNA. The final concentration was made to 20 ng/μl and stored at −20 °C until further use. A total of 30 random decamer primers (Genemed Synthesis Inc., TX, USA) were screened for RAPD analysis, out of which 12 primers were selected on the basis of clarity of banding patterns. The protocol for RAPD analysis was adapted from that of Williams et al. [22] with little modification. DNA amplification was performed with reaction volume of 25 μl containing 10× PCR buffer (2.5 μl), 2.5 mM MgCl_2_ (1.5 μl), 100 mM dNTPs (2.0 μl), primer (2.0 μl), Taq polymerase (0.2 μl), 40 ng of template DNA (2.0 μl), and 14.8 μl sterile D.W. PCR was performed at initial temperature of 94 °C (6 min, 1 cycle), followed by 38 cycles of 30 s at 94 °C, 30 s at 36 °C and 2 min at 72 °C, and a final cycle of 10 min at 72 °C.

### 2.6 Statistical analysis

Experiments were set up in a completely randomized block design and each experiment repeated thrice with 20 tubes per treatment. Comparison between the mean values of treatments was made using Dunnett multiple comparison test (DMCT) at 0.05 and 0.01 levels of significance. For RAPD analysis, each amplified product was scored as present (1) or absent (0) and the similarity matrix was obtained by Jaccard coefficient using NTSYS software.

## 3 Results and discussion

### 3.1 Seed germination and shoot proliferation

The present investigation on micropropagation technique was established for the first time in *S. lawii*. This technique has previously been successfully used in many economically important and threatened plants due to its simplicity, low risk of genetic instability and high propagation rate [15], [16]. *In vitro* seed germination was successfully achieved on MS medium devoid of plant growth regulators. The effectiveness of cytokinins and their combinations with auxins were tested for shoot multiplication from shoot tip explants of *in vitro* germinated seedlings. Various morphogenic responses obtained with different concentrations and combinations of PGRs are summarized in Table 1. Significant differences were observed in tested concentrations and combinations of cytokinins and auxins (Table 1). MS medium lacking PGRs failed to induce the shoots. Individually, BAP proved to be most efficient (100% response) and produced an average of 6.8 ± 0.4 shoots and 3.6 ± 0.2 cm shoot length in 3.0 mg l^−1^ concentration. Previously, enhanced shoot multiplication in the presence of BAP has been reported in *S. chirayita* [16]. Maximum shoot multiplication (100%) and highest shoot number (10.4 ± 0.8) was obtained in MS medium supplemented with a combination of 3.0 mg l^−1^ TDZ and 0.3 mg l^−1^ IBA (Table 1, Fig. 1b and c). However, a combination of BAP (3.0 mg l^−1^) and IBA (0.3 mg l^−1^) served better for shoot length (5.9 ± 0.3 cm). A synergistic effect of cytokinins and auxins on increased shoot multiplication was well noticed in many threatened medicinal plants including *Swertia* species [18], [19], [23].

**Table 1:** Effect of different plant growth regulators on shoot regeneration from shoot tip explants of *Swertia lawii*.

PGRs	Concentration (mg l^−1^)	Regeneration frequency (%)	Number of shoots/explant (mean ± SE)	Length of shoots (cm) mean ± SE
PGR free	00	00	0.0	0.0
BAP	1.0	100	6.3 ± 0.3**	3.3 ± 0.3**
	2.0	100	4.8 ± 0.4**	2.4 ± 0.1**
	3.0	100	6.8 ± 0.4**	3.6 ± 0.2**
	4.0	100	6.1 ± 0.4**	4.7 ± 0.5**
	5.0	100	6.2 ± 0.4**	2.7 ± 0.1**

KN	1.0	100	4.5 ± 0.2**	2.9 ± 0.1**
	2.0	100	4.5 ± 0.3**	2.8 ± 0.2**
	3.0	95	3.0 ± 0.2**	2.3 ± 0.2*
	4.0	90	3.2 ± 0.3**	2.8 ± 0.4**
	5.0	90	2.9 ± 0.2**	2.2 ± 0.2*

TDZ	1.0	60	1.7 ± 0.3*	0.7 ± 0.2^ns^
	2.0	85	3.1 ± 0.4**	2.7 ± 0.5**
	3.0	100	5.8 ± 0.5**	3.0 ± 0.2**
	4.0	95	5.7 ± 0.8**	1.8 ± 0.1*
	5.0	95	5.4 ± 0.6**	2.1 ± 0.2*

BAP + IBA	3.0 + 0.1	100	6.8 ± 0.4**	3.5 ± 0.1**
	3.0 + 0.3	100	7.1 ± 0.3**	5.9 ± 0.3**
	3.0 + 0.5	100	6.9 ± 0.4**	3.4 ± 0.2**
	3.0 + 0.7	100	6.4 ± 0.4**	2.9 ± 0.1**
	3.0 + 1.0	100	6.7 ± 0.6**	2.3 ± 0.2*

TDZ + IBA	3.0 + 0.1	100	5.9 ± 0.7**	2.3 ± 0.2*
	3.0 + 0.3	100	10.4 ± 0.8**	3.7 ± 0.4**
	3.0 + 0.5	100	8.1 ± 0.8**	3.9 ± 0.3**
	3.0 + 0.7	100	5.9 ± 0.4**	3.2 ± 0.2**
	3.0 + 1.0	100	5.7 ± 0.3**	2.7 ± 0.1**

Mean ± S.E. of 30 replicates per treatment and experiments were repeated thrice. The values are significantly different at ns – non significant, *P < 0.05 and **P < 0.01 level when compared by Dunnett multiple comparisons test using one way ANOVA.

### 3.2 Callus induction and cell suspension cultures

**Fig. 2:**
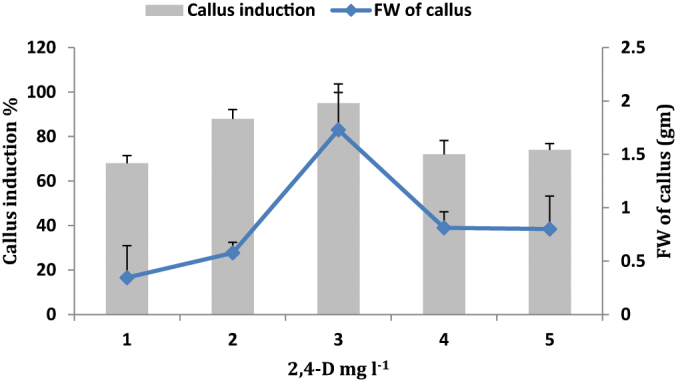
Effect of various concentrations of 2, 4-D on callus induction and fresh weight of callus in *Swertia lawii*.

**Fig. 3:**
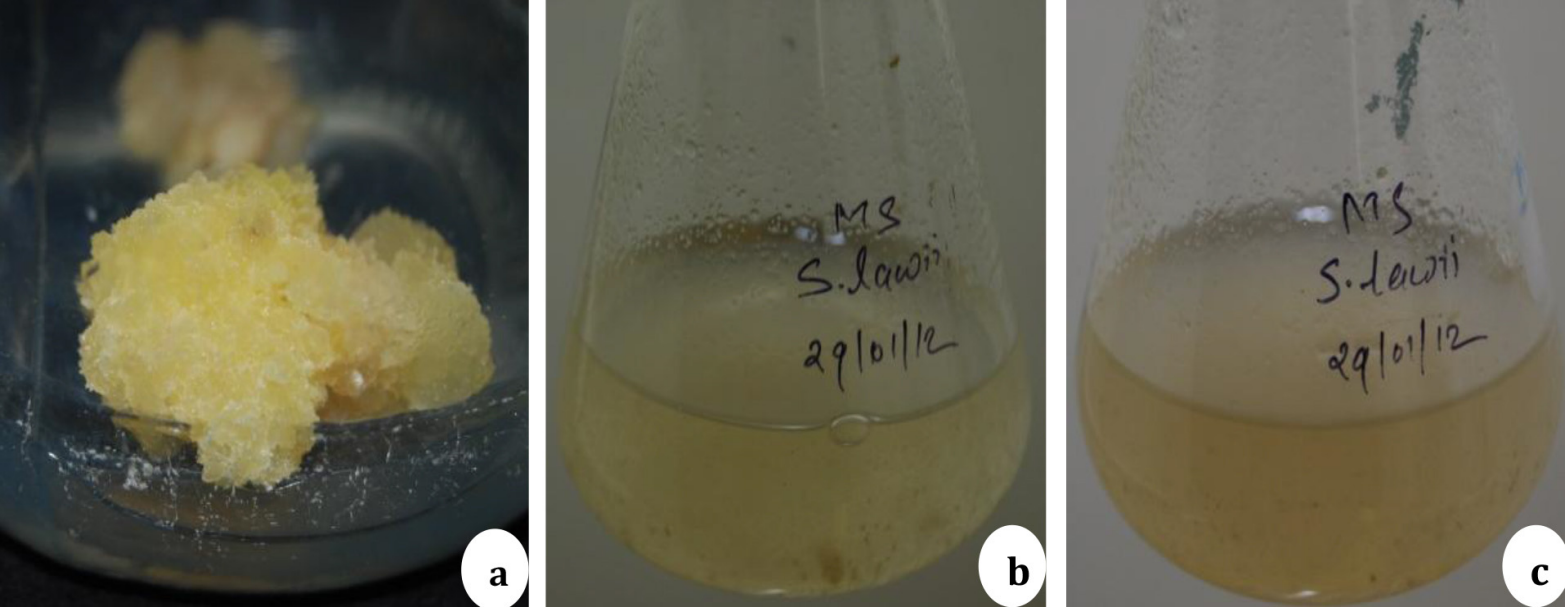
Callus and cell suspension cultures of *Swertia lawii*. a: Callus induction and proliferation (MS + 2.0 mg l^−1^ 2,4-D), b: cell suspension after 15 days (2.0 mg l^−1^ BAP + 2.0 mg l^−1^ 2,4-D) c: cell suspension after 30 days (2.0 mg l^−1^ BAP + 2.0 mg l^−1^ 2,4-D).

The effects of different concentrations of 2, 4-D (1.0–5.0 mg l^−1^) were tested for induction and proliferation of callus from leaf explants (Fig. 2). A wide range of variation was observed in percentage of callus induction and fresh weight of callus (Fig. 2). MS medium without PGRs failed to induce the calli. Highest frequency of callus induction (95 ± 4.8%) and maximum fresh weight of callus (1.7 ± 0.4 gm) was observed in the MS medium supplemented with 2, 4-D (3.0 mg l^−1^). In the same medium, whitish and friable callus was developed (Fig. 3a). The inclusion of 2, 4-D in the culture medium has been reported to be necessary for inducing callus in *S. chirayita* [24], *Gentiana straminea* [25], and *Swertia mussotii* [20].

**Fig. 4:**
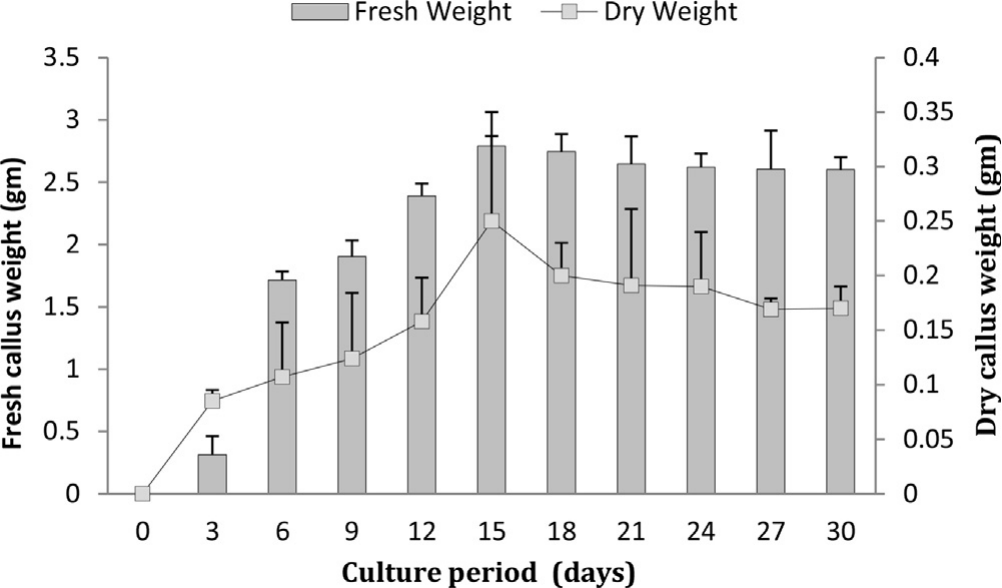
Growth kinetics of cells in suspension cultures of *Swertia lawii* supplemented with 2, 4-D (2.0 mg l^−1^) and BAP (2.0 mg l^−1^).

Cell suspension culture technology has immense importance in the production of secondary metabolites [26]. *In vitro* production of secondary metabolite in plant cell suspension cultures has been reported from various medicinal plants including *S. chirayita* [27]. However, not much study has been carried out with other *Swertia* species. In the present investigation, cell suspension cultures were established by culturing calli developed on 2, 4-D (2.0 mg l^−1^) supplemented media and inoculated in liquid MS fortified with different concentrations and combinations of BAP, 2, 4-D and glutamine (200 mg l^−1^). MS liquid medium supplemented with 2, 4-D (2.0 mg l^−1^) and BAP (2.0 mg l^−1^) showed well established suspension cultures in which no aggregation or clumps of cells were observed (Fig. 2b and c). This medium is used for further growth kinetics study and is summarized in Fig. 4. Cells in suspension showed the better growth for first two weeks and there after the reduction in fresh as well as dry weight of callus was observed (Fig. 4). The maximum growth of cells (fresh weight = 2.8 g and dry weight 0.6 g) was observed at 15 day of culture in MS liquid medium supplemented with 2, 4-D (2.0 mg l^−1^) and BAP (2.0 mg l^−1^). Similarly, maximum growth of cells in suspension were observed between 10–15 days were reported in *Passiflora alata* [28].

### 3.3 Rooting and hardening of in vitro raised plantlets

For *in vitro* rooting, regenerated shoots were isolated from the culture and placed on MS medium supplemented with different concentrations of auxins *viz*. IBA, IAA and NAA (Table 2). The rooting percentage, number of roots and root length were recorded after 4 week of culture. In the present study, the best rooting frequency (100%), maximum number of roots (22.1 ± 2.5) and highest root length (2.5 ± 0.3 cm) was achieved on MS medium supplemented with 3.0 mg l^−1^ IAA (Table 2, Fig. 1c). In contrast, microshoots of *S. chirayita* rooted best in MS medium supplemented with various concentrations of NAA and IBA [17], [18]. In this study, both NAA and IBA produced significant numbers of roots; however they were less as compared to IAA. Increased concentrations (over 4.0 mg l^−1^) of IBA and NAA showed decreased number of roots (Table 2). Well rooted plantlets from optimal rooting media were washed to eliminate the adhering culture medium and successfully transferred in to the plastic pots containing a mixture of sterilized soil, sand and coco peat in 1:2:1 ratio (Fig. 1e). Two month after transferring, the survival rate of the plantlets was highest (90%) and the plants grew vigorously and exhibited true characters similar to the field grown ones and could be transplanted at their natural habitats. Similar results were reported where increased quantity of sand significantly enhanced the survival rate of micropropagated *Ceropegia panchganiensis* [15].

**Table 2:** Effect of different auxins on *in vitro* rooting of microshoots of *Swertia lawii*.

PGRs	Concentration (mg l^−1^)	Regeneration frequency (%)	Number of shoots (mean ± SE)	Length of shoots (cm) (mean ± SE)
PGR free	00	00	0.0	0.0
IBA	1.0	100	14.7 ± 2.8**	1.7 ± 0.3**
	2.0	100	16.8 ± 1.8**	1.4 ± 0.1**
	3.0	95	16.7 ± 2.4**	0.8 ± 0.2**
	4.0	80	08.6 ± 1.7*	0.4 ± 0.1^ns^
	5.0	65	10.0 ± 2.3**	0.3 ± 0.1^ns^
IAA	1.0	100	15.5 ± 1.2**	1.5 ± 0.1**
	2.0	100	12.8 ± 0.8**	1.8 ± 0.2**
	3.0	100	22.1 ± 2.5**	2.5 ± 0.3**
	4.0	100	21.2 ± 1.8**	2.4 ± 0.1**
	5.0	95	15.4 ± 1.5**	2.1 ± 0.1**
NAA	1.0	70	14.6 ± 2.9**	1.5 ± 0.2**
	2.0	75	13.2 ± 3.1**	1.0 ± 0.2**
	3.0	70	11.3 ± 2.6**	0.8 ± 0.2**
	4.0	60	09.9 ± 2.6*	0.5 ± 0.1^ns^
	5.0	65	07.2 ± 2.0^ns^	0.4 ± 0.1^ns^

Mean ± S.E. of 30 replicates per treatment and experiments were repeated thrice. The values are significantly different at ns – non significant, *P < 0.05 and **P < 0.01 level when compared by Dunnett multiple comparisons test using one way ANOVA.

### 3.4 Genetic fidelity analysis

**Fig. 5:**
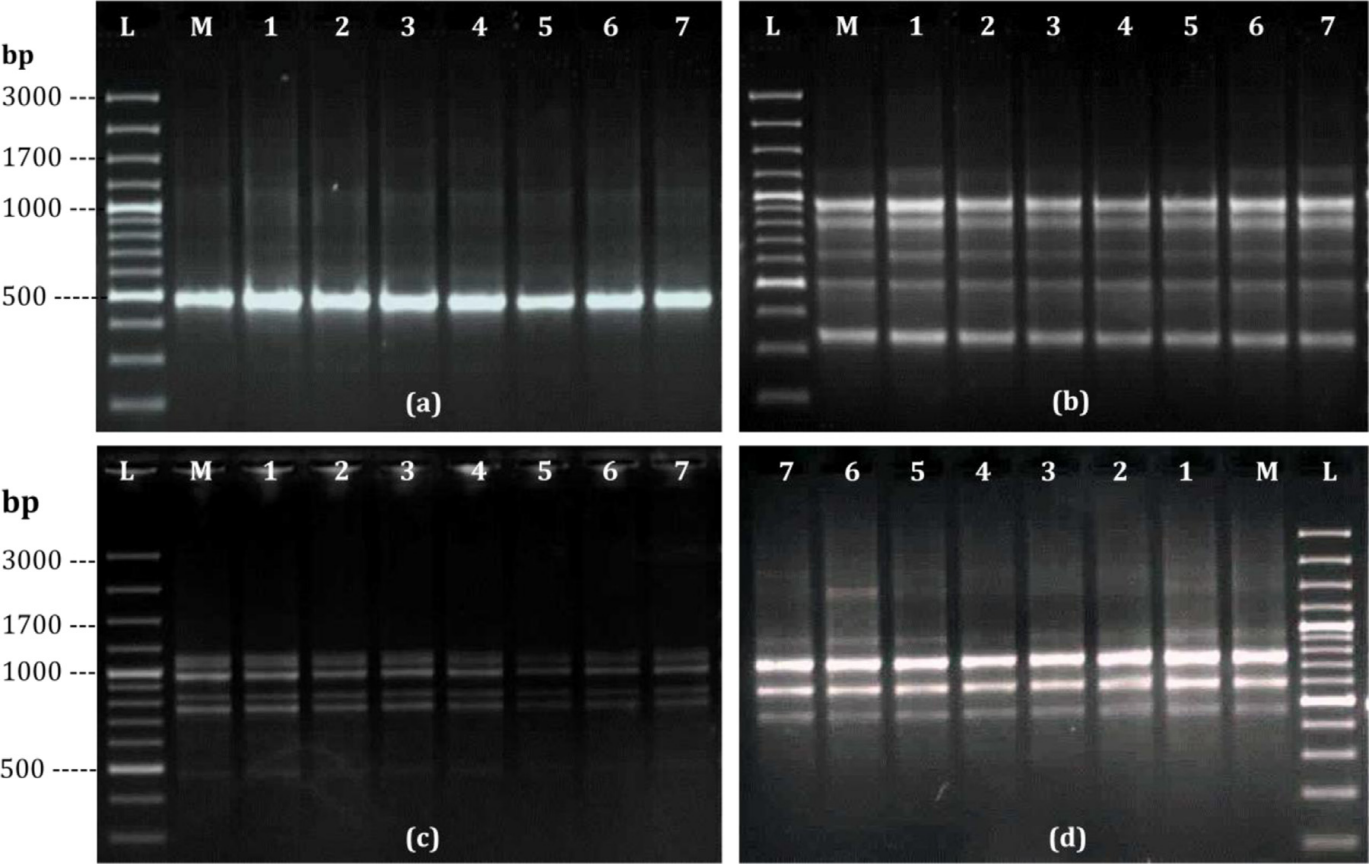
DNA fingerprinting patterns generated with RAPD primers. a: OPV-10, b: OPW-16, c: OPL-05, d: OPV-19, among mother (M) and regenerants (lane 1–7), molecular weight marker (lane L), : polymorphic band.

The main aim of *in vitro* regeneration is to produce the true to type plants, therefore it is required to confirm the genetic fidelity of *in vitro* raised clones. The DNA based marker techniques are very much important to assure the genetic fidelity of micropropagated plants [15], [16], [29]. A total of 30 RAPD primers were used for initial screening with the mother as well as seven randomly selected micropropagated plants of *S. lawii* but only 12 RAPD primers gave clear and reproducible bands which were used for further analysis. The number of scorable bands for each RAPD primer varied from 1 (OPV-10) to 9 (OPF-04) (Table 3). The 12 RAPD primers produced 49 distinct and scorable bands, with an average of 6.1 bands per primer. Each primer generated a unique set of amplification products ranging in size from 200 bp (OPD-12, OPD-13) to 1200 bp (OPF-04). A total of 405 bands were generated with 12 RAPD primers in a mother plant and 7 micropropagated plants, out of which 400 (98.76%) bands were monomorphic and rest 5 (1.24%) were polymorphic (Table 3). In other words, the micropropagated plantlets showed high monomorphism with few polymorphic bands (Fig. 5a–d). RAPD primer OPV-19 showed single polymorphic band in clone-6 (Fig. 5d), while primer OPV-18 showed 4 additional bands in clone-6 and clone-7. A variation during micropropagation depends upon the source of explants and the mode of *in vitro* regeneration [30], [31]. In the present study, high levels of uniformity among donor and *in vitro* raised clones was observed. Similarly high levels of monomorphism were also reported in threatened medicinal plants *viz*. *C. panchganiensis* [15] and *Arnebia hispidissima* [29]. These findings support the fact that a shoot apices or meristem-based micropropagation system is much more stable genetically than those in which regeneration occurs *via* the callus phase.

**Table 3:** List of primers, their sequences, number and size of the amplified fragments generated by RAPD primers in *Swertia lawii*.

Sr. no.	Primer code	Primer sequence (5′–3′)	Number of bands/primer	Total number of bands amplified		Size range (bp)
				Monomorphic bands	Polymorphic bands	
1	OPD-12	CACCGTATCC	5	40	–	200–1000
2	OPD-13	GGGGTGACGA	3	24	–	200–700
3	OPF-04	GGTGATCAGG	9	72	–	500–1200
4	OPH-04	GGAAGTCGCC	5	40	–	500–900
5	OPL-03	CCAGCAGCTT	3	24	–	600–900
6	OPL-05	ACGCAGGCAC	5	40	–	450–1075
7	OPT-05	GGGTTTGGCA	4	32	–	525–1000
8	OPV-10	GGACCTGCTG	1	08	–	475–500
9	OPV-18	TGGTGGCGTT	4	32	4	375–1000
10	OPV-19	GGGTGTGCAG	4	32	1	450–1000
11	OPW-16	CAGCCTACCA	5	40	–	300–950
12	OPW-19	CAAAGCGCTC	2	16	–	275–1000

Total			49	400	5	

## 4 Conclusion

A simple and effective micropropagation system has been developed for *S. lawii* by using shoot tip explants. To our knowledge this is the first report on *in vitro* regeneration, callus and cell suspension cultures in *S. lawii*. Moreover, the genetic uniformity of regenerants was assessed by RAPD markers. This protocol imparts successful and rapid technique that can be utilized for the commercial propagation, *ex situ* conservation and genetic transformation of this medicinal herb for its further improvement. Establishment of callus and cell suspension cultures will provides new strategy for the enhancement of secondary metabolites.
